# Genetic diversity evaluation of *Luculia yunnanensis*, a vulnerable species endemic to Yunnan, Southwestern China based on morphological traits and EST-SSR markers

**DOI:** 10.3389/fpls.2024.1428364

**Published:** 2024-08-14

**Authors:** Yao Zhang, Youming Wan, Suping Qu, Zhengchun Mu, Yuying Li, Zhenghong Li

**Affiliations:** ^1^ Institute of Highland Forest Science, Chinese Academy of Forestry, Kunming, China; ^2^ College of Forestry, Nanjing Forestry University, Nanjing, China; ^3^ Flower Research Institute, Yunnan Academy of Agricultural Sciences, Kunming, China; ^4^ Department of Ecological Protection and Restoration, Gongshan Forestry and Grassland Administration, Gongshan, China; ^5^ College of Grassland Science, Shanxi Agricultural University, Jinzhong, China

**Keywords:** *Luculia yunnanensis*, morphological characteristics, EST-SSR markers, genetic diversity, genetic differentiation, genetic structure, conservation genetics

## Abstract

*Luculia yunnanensis* is a vulnerable species endemic to Yunnan Province, Southwestern China, which has high ornamental value. Its wild population has not been fully protected and utilized for a long time, which is not conducive to the long-term stable development of this species. Genetic diversity assessment is the basis and prerequisite for the conservation of rare species. In this study, 21 phenotypic traits and 17 highly polymorphic EST-SSR markers were used to analyze the genetic diversity and genetic structure of 164 individuals from six *L. yunnanensis* populations. The coefficient of variation of 21 phenotypic traits ranged from 11.76% to 52.58% (mean=21.72%), and the coefficient of variation of 18 traits was less than 30%. The average values of *Ne*, *I*, *Ho* and *He* were 1.710, 0.619, 0.384, and 0.352, respectively. The genetic diversity of LLO (*Ho* = 0.476 and *He* = 0.426) and LCM (*Ho* = 0.424 and *He* = 0.381) populations in Lushui County was highest. The GDX populations (*Ho* = 0.335 and *He* = 0.269) isolated by Gaoligong Mountain had the lowest genetic diversity. The AMOVA results showed that 13.04% of the genetic variation was among populations and 86.96% was within populations. The average phenotypic differentiation coefficient of phenotypic traits among populations was 18.69%. The results of phenotypic and genetic variation analysis were consistent, indicating that the most of variation exists within population. Genetic structure, UPGMA clustering and PCA analysis results showed that the populations of *L. yunnanensis* had obvious geographical divisions, and the populations distributed in the southern region and distributed in the northern region of the Nujiang River clustered into one group respectively. Combining the results of phenotypic and molecular markers, we recommend that give priority to the protection of LLO, LCM and GDX population, in order to ensure the sustainable utilization of *L. yunnanensis* germplasm resources.

## 1 Introduction

*Luculia yunnanensis* (*Luculia*) is an evergreen shrub or small tree, endemic to Yunnan province, Southwestern China. It mainly grows on the hillside, understory or in the thickets at altitude of 1200-3200m. *L. yunnanensis* is a type of woody flower with high development and utilization value owing to its beautiful plant shape, long flowering period, chromatic color and attractive aroma. According to the records, this species is distributed in Gongshan, Fugong, Lushui, Jingdong, Zhenkang, Luxi, and Motuo counties. However, in the previous investigation of our research team, it was found that its distribution range showed an obvious shrinking trend. It only exists in Lushui, Fugong and Gongshan Counties, and the distribution area is 10,465.48 km^2^, only 20% of its historical range (Li, 2017; Zhang et al., 2022). According to the IUCN (International Union for Conservation of Nature) threat level criteria, this species belongs to the vulnerable species category (Li, 2017). Therefore, the wild resource of *L. yunnanensis* is in urgent need of conservation.

At present, little research on *L. yunnanensis* was reported. Wan et al. (2010) studied the seed germination character and optimal conditions of *L. yunnanensis*. Zhou et al. (2010) developed 13 pairs of SSR primers suitable for analyzing the genetic diversity of *Luculia*, which could effectively distinguish between *L. yunnanensis* and *Luculia pinceana*. Ma et al. (2012) made use of a modified biotin–streptavidin capture method to develop 11 pairs of SSR primers with polymorphism in two populations of *L. yunnanensis*. Li et al. (2017) determined the floral components of *L. yunnanensis*. Zhang et al. (2022) developed 17 EST-SSR primers with polymorphism in six populations of *L. yunnanensis* based on transcriptomic data. Up to now, there have been no reports on the analysis and evaluation of genetic diversity of *L. yunnanensis* population.

Genetic diversity is the product of long-term evolution of biological population, and is the premise of its survival and development. For a species, the higher the genetic diversity, the richer the genetic variation, and the better the ability to adapt to environmental changes. Research on genetic diversity can provide theoretical basis and guidance for resource evaluation, conservation and utilization of species. Before the conservation of rare plants, it is necessary to identify the genetic diversity of threatened species and some relevant factors should be taken into account, so as to propose effective and feasible conservation measures. The research methods of genetic diversity include morphological, cytological, biochemical and molecular marker (Pop et al., 2013; Xu et al., 2016; Li et al., 2022a; Jia et al., 2024). Among them, morphology is the most simple and convenient marker method, which can reveal the degree of genetic variation and explore excellent germplasm to a certain extent. Morphological traits can be greatly influenced by the ecological conditions, and they are not fully effective in determining species diversity (Zhu et al., 2022). However, molecular markers are not affected by ecological factors, which can make up for the deficiency of morphological markers. In view of the advantages and disadvantages of morphological and molecular markers, they can be combined to study the genetic diversity of species (El Kadri et al., 2019; Li et al., 2023a; Sivan et al., 2023; Nivedha et al., 2024; Zhang et al., 2024). EST-SSR markers are one of the most widely used genetic markers with advantages such as simple operation, good stability, high accuracy, high repeatability and good interspecific transmission (Wang et al., 2021; Li et al., 2022b).

In view of this, 21 morphological traits and 17 EST-SSR molecular markers were used to analyze the genetic diversity and genetic structure of six *L. yunnanensis* wild populations. The objective is to understand the population structure and genetic background of *L. yunnanensis*, so as to provide reference and basis for its resource conservation, germplasm screening and marker-assisted breeding.

## 2 Materials and methods

### 2.1 Plant materials

**Figure 1:**
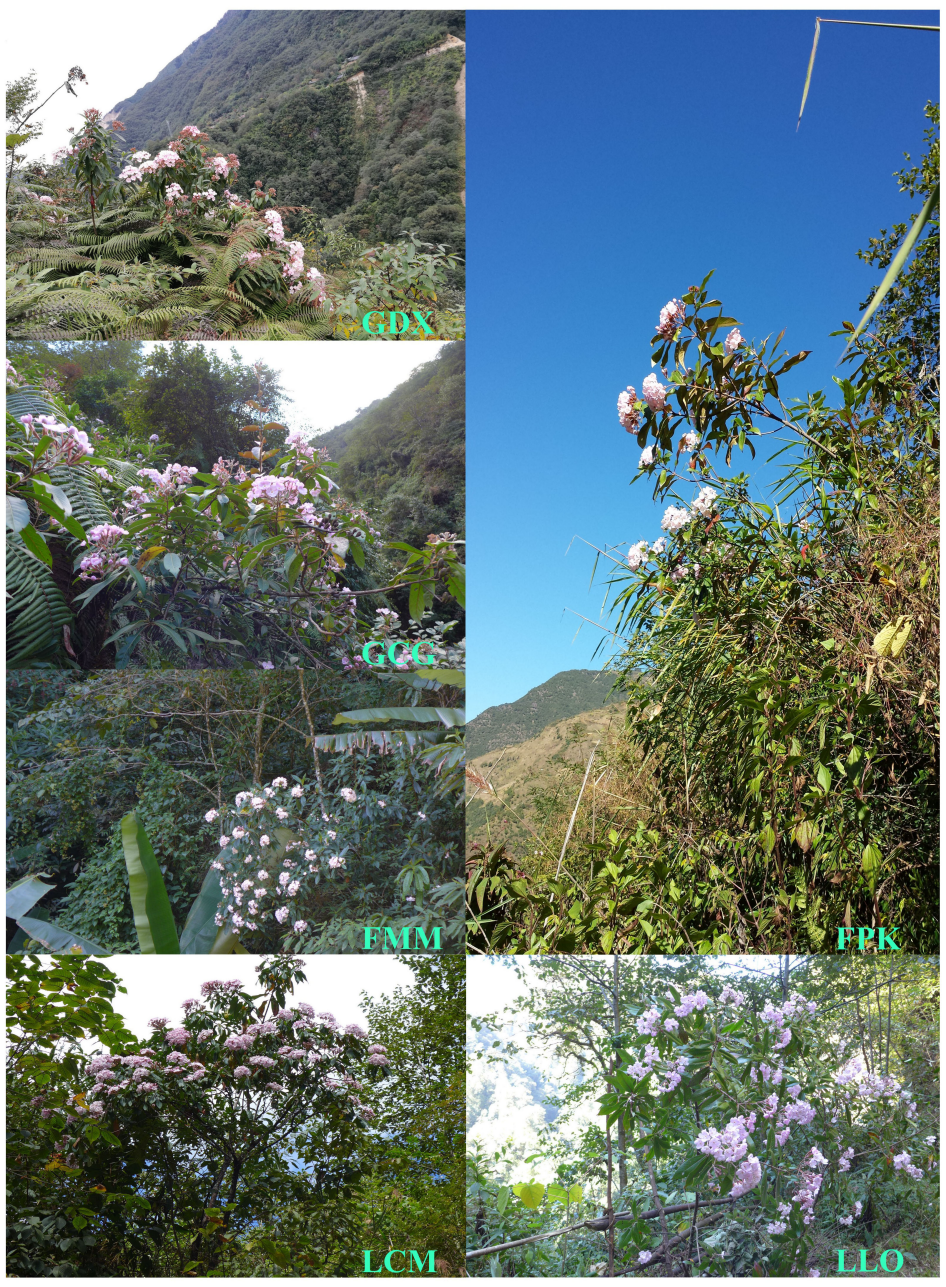
Geographic distribution of six *L. yunnanensis* populations.

**Figure 2:**
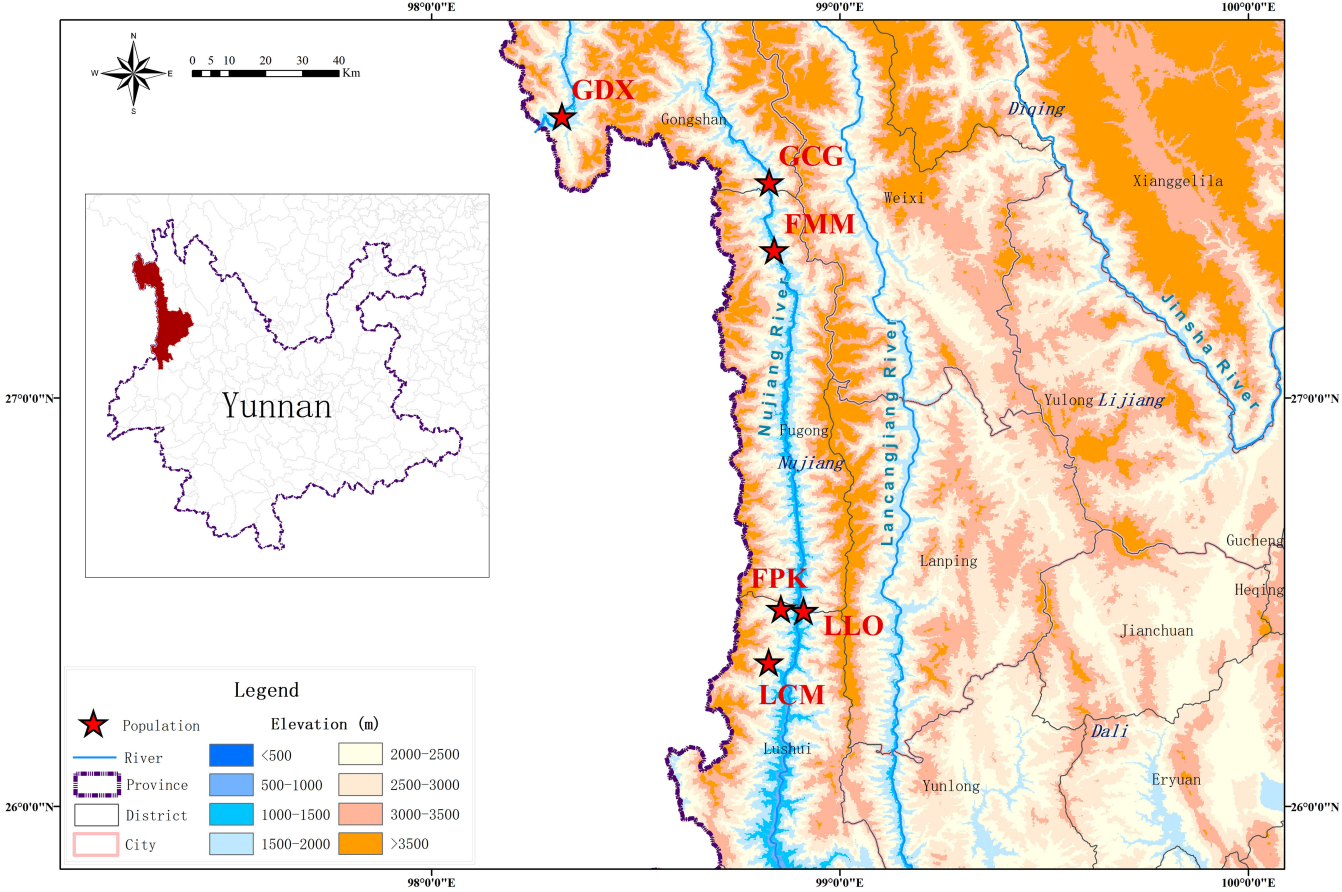
Habitat of six *L. yunnanensis* populations.

According to the preliminary resource survey, *L. yunnanensis* only exists in Lushui, Fugong and Gongshan counties in Nujiang Prefecture, Yunnan Province. One population was found on the west side of Gaoligong Mountain. The remaining populations were distributed in the altitude from 1300 to 2200 meters of the Nujiang River Valley on the east side of Gaoligong Mountain and west side of Biluo Snow Mountain. Considering the influence of mountains, rivers and geographical distance on the genetic diversity, we selected 164 individuals from six representative populations of *L. yunnanensis* as research objects for phenotypic survey and molecular experiments. Each population was randomly surveyed with 20-30 mature plants, and to minimize the relationship between plants, the spacing between research individuals was not less than 10 m. The locations and habitats of six *L. yunnanensis* populations are shown in **Table 1**, **Figures 1**, **2**, which are GDX, GCG, FMM, FPK, LLO and LCM.

**Table 1:** Information on sampled populations of *L. yunnanensis*.

Population	Location	Longitude	Latitude	Altitude
GDX	Dulongjiang township, Gongshan County	98.3188−98.3466	27.6915−27.9160	1324–1788
GCG	Cikai town, Gongshan County	98.6088−98.8334	27.5254−27.7472	1325–1778
FMM	Maji township, Fugong County	98.8388−98.8453	27.3632−27.3651	1354–1455
FPK	Pihe township, Fugong County	98.9102−98.9106	26.4799−27.4801	1738–1752
LLO	Luobenzhuotownship, Lushui County	98.8116−98.8172	26.4780−26.4812	2029–2153
LCM	Chenggantownship, Lushui County	98.8253−98.8312	26.3540−26.3563	1664–1736

### 2.2 Phenotypic survey

A total of 21 morphological traits were investigated from three aspects: flower characteristics, leaf morphology and fruit morphology. 16 flower traits: select plants that are in full-bloom stage, and one inflorescence was selected from the middle, east, west, south and north of the crown periphery for investigation. Three leaf traits: five healthy leaves with physiological functions were randomly selected for the investigation of leaf shape and petiole length. Two fruit traits: five fully mature capsules were randomly selected, and their fruit length and width were measured. Among the 21 indexes, qualitative traits included heterostyly, flower color and bud color. The qualitative traits were based on direct observation, with reference to researchers’ descriptions and evaluations of phenotypic traits of other ornamental plants (Chen et al., 2022; Zhou et al., 2024; Li et al., 2023b), and standardized to assign values. Heterostyly: short style recorded as 1 and long style as 2. Flower and bud color: in natural light, observe the petal color when the corolla is fully expanded (bloom) and when the corolla is closed (buds). The part that involves the flower color we use the Royal Horticultural Society Color Chart (RHSCC, 2015 version) for observation and comparison. Assign a value standard: 0= white, 1= white with light pink (white + 56C), 2= white with pink (white + 55C), 3= light pink (56C), 4= pink (55C), 5= dark pink (55B), 6= red (63B). 18 quantitative traits were measured with straightedge or vernier caliper, of which inflorescence diameter was measured by straightedge and the remaining traits were measured by vernier caliper. The measurement results were averaged for calculation and analysis.

### 2.3 DNA extraction and SSR primer information

Total genomic DNA of *L. yunnanensis* was extracted from the leaves using a plant genomic DNA extraction kit containing RNase A (TransGen, Beijing, China) following the manufacturer’s protocol. The quality and quantity of extracted genomic DNAs were checked by 0.8% agarose gel electrophoresis and determined using a NanoDrop 2000 spectrophotometer (Thermo Fisher Scientific, Wilmington, DE, USA), respectively. The high-quality DNAs were stored at -20°C refrigerator until further use. All samples were genotyped using 17 EST-SSR markers previously developed for the species (Zhang et al., 2022). The 17 pairs of EST-SSR primers were synthesized by Kunming Shuoqing Biotechnology Co., Ltd.

### 2.4 PCR amplification and product detection

PCR amplification system and procedure had been reported previously by Zhang et al. (2022). Briefly, the 10-μL reaction mixture included 10-30 ng of genomic DNA, 0.6 mmol/L each of reverse and forward primer, 10*×*Taq buffer, 0.15 mmol/L of each dNTP, and 1 unit of Taq DNA polymerase (TransGen, Beijing, China). Amplification was performed in a Bio-Rad C1000 Touch ™ Thermal Cycler (Bio-Rad Laboratories,CA, USA), and the program was as follows: 5 min at 94°C, 30 cycles (30 s at 94°C, annealing at 50~60°C for 30 s, 1 min at 72°C), and a final extension at 72°C for 10 min. SSR primers were modified with two fluorescent markers (FAM or HEX) at 5′-end, and multiplex PCR amplification was performed using the above PCR conditions. The PCR products were separated and visualized with ABI 3730xl DNA Analyzer (Thermo Fisher Scientific, Wilmington, DE, USA), and allele sizes were assessed using the GeneMapper 4.1 (Thermo Fisher Scientific, Wilmington, DE, USA).

### 2.5 Data analysis

SAS software (SAS Institute, Cary, NC, USA, 1990) was used to variance analysis of morphological characters (Li et al., 2023a), and Duncan’s test was used for multiple comparisons. SPSS was used to calculate Pearson correlation coefficient to analyze the correlation of 21 morphological traits. Phenotypic data were collated and standardized by Excel and SPSS. The following parameters of genetic diversity were calculated by the GenAlEx v.6.5 (Peakall and Smouse, 2012): number of alleles (*Na*), effective number of alleles (*Ne*), Shannon’s information index (*I*), observed heterozygosity (*Ho*), expected heterozygosity (*He*). Nei’s genetic identities (GI) and genetic distances (GD), private alleles (*P_AS_*), F-statistics (inbreeding coefficients, *F_is_* and genetic differentiation coefficient, *F_ST_*) were also calculated using this software, and gene flow (*Nm*) was estimated using Wright’s method, *Nm* =0.25(1 - *F_ST_*)/*F_ST_)* (Wright, 1931; Rousset, 1997). Meanwhile, principal component analysis (PCA) based on genetic distance was conducted using GenAlEx v.6.502 (Wang et al., 2011; Moghim et al., 2012). Origin software was used to carry out the PCA of the phenotypic traits (Zhou et al., 2024). Analysis of molecular variance (AMOVA) was carried out to estimate the distribution of genetic variation among and within the populations using ARLEQUIN version 3.0 (Excoffier et al., 2005). UPGMA (unweighted pair group method with an arithmetic mean) cluster analysis was performed by NTSYSpc 2.1 (Rohlf, 2000). The polymorphic information content (PIC) was calculated by using PIC_CALC 0.6 software (Sándor et al., 2012). Population structure of all individuals in different populations were inferred by the Bayesian clustering method in STRUCTURE 2.3.4 (Pritchard et al., 2000; Evanno et al., 2005) Operation parameter setting: length of burn-in period and number of Markov chain Monte Carlo (MCMC) repeats after burn-in were set at 30000 and 100000, twenty independent runs were made with values of K set from 1 to 6. ΔK was calculated by a web-based tool Structure Selector (https://lmme.ac.cn/StructureSelector/).

## 3 Results

### 3.1 Morphological characteristics analysis

In this study, 21 phenotypic traits were observed and analyzed. The results of correlation analysis showed that there were significant correlations between most of morphological traits of *L. yunnanensis* germplasm resources (**Table 2**). A total of 102 pairs of traits showed highly significant correlation, and 16 pairs showed significant correlation. The three leaf traits (petiole length, leaf length, and leaf width) showed highly significant positive correlations between each pair, and these three traits showed highly significant or significant correlations with all the other flower traits except for a few traits related to style and petal color. The correlation between fruit length and fruit width were significant, however they were not obviously related to the leaf and flower traits. Among the 16 flower traits, there was a highly significant positive correlation between flower color and bud color, and both of them were significantly negatively correlated with anther length and width. Anthotaxy diameter had no correlation with heterostyly, stigma lobes length, petal color and fruit, but had significant correlation with the other 14 morphological traits. Among them, the correlation between anthotaxy diameter and flower number in an anthotaxy was the highest. The results of variance analysis (**Table 3**) showed that there were no differences in style type, sepal width, style length, anther length and fruit width between populations. The flower color was redder at the bud stage than that at the flowering stage, and the flower color of the LLO population was redder than that of the other populations. The GDX and GCG populations had the longest pedicel length. The corolla width of GDX, GCG and FMM populations were larger than those of the other three populations. The anthotaxy diameter, flower number in an anthotaxy, leave length and leave width were the largest in GDX population and the smallest in LCM population. The LLO and LCM populations had shorter corolla tube lengths and the smallest anther width than the other populations. The coefficient of variation of phenotypic traits reflects the variation status among samples and reveals the variation pattern of populations (Zhang et al., 2012). A higher coefficient of variation indicates a greater dispersion of phenotypic traits. It can be seen from **Table 4** that among the 21 phenotypic traits in the populations of *L. yunnanensis*, the largest coefficient of variation is the flower number in an anthotaxy (52.58%), and the smallest is the corolla tube length (11.76%). The coefficient of variation of 18 phenotypic traits indexes was between 10% and 30%, indicating that the variation degree of phenotypic traits was relatively stable. The phenotypic variation coefficient of the LLO population (24.93%) was slightly higher than that of the other five populations, and the phenotypic variation coefficient of the other populations was in the range of 17.21% to 21.20%. According to the variance components and differentiation coefficients within and among populations, leaf length showed the greatest phenotypic differentiation degree among populations (68.35%), followed by peduncle length (63.84%) and flower number in an anthotaxy (50.24%). The average phenotypic differentiation coefficient of 21 phenotypic traits among populations was 18.69%, indicating that the phenotypic differentiation degree among populations was not high, and most of the phenotypic trait differentiation came from within populations.

**Table 2:** Pearson correlation coefficient of the morphological traits.

	C1	C2	C3	C4	C5	C6	C7	C8	C9	C10	C11	C12	C13	C14	C15	C16	C17	C18	C19	C20	C21
C1	1																				
C2	.131	1																			
C3	-.012	.687**	1																		
C4	-.018	.088	.035	1																	
C5	.050	.076	.015	.731**	1																
C6	-.021	.024	-.079	.647**	.460**	1															
C7	-.066	-.025	.032	.308**	.167*	.474**	1														
C8	-.100	.049	.102	.396**	.117	.391**	.328**	1													
C9	-.107	.041	.079	.350**	.156*	.418**	.304**	.636**	1												
C10	-.197**	-.070	-.110	.411**	.131	.378**	.274**	.614**	.461**	1											
C11	-.540**	-.165*	-.047	.384**	.060	.280**	.326**	.490**	.392**	.579**	1										
C12	.703**	.098	.009	.311**	.113	.238**	.181*	.274**	.203**	.230**	.039	1									
C13	-.851**	-.172*	-.041	.034	-.086	.031	.148*	.202**	.158*	.287**	.642**	-.497**	1								
C14	-.852**	-.174*	-.053	.226**	.013	.152*	.210**	.322**	.259**	.442**	.825**	-.341**	.835**	1							
C15	-.030	-.148*	-.202**	.331**	.129	.349**	.327**	.458**	.326**	.518**	.474**	.319**	.191*	.314**	1						
C16	-.255**	-.252**	-.320**	.238**	.038	.304**	.275**	.301**	.259**	.495**	.464**	.037	.401**	.426**	.709**	1					
C17	-.085	.108	.069	.622**	.456**	.608**	.323**	.420**	.390**	.398**	.340**	.146	.071	.216**	.335**	.277**	1				
C18	-.027	.093	.034	.558**	.425**	.511**	.192*	.309**	.344**	.364**	.271**	.176*	.027	.156*	.304**	.245**	.848**	1			
C19	.030	.053	-.039	.489**	.342**	.472**	.229**	.406**	.285**	.379**	.256**	.205**	.002	.133	.377**	.307**	.733**	.625**	1		
C20	-.060	-.036	-.010	.158	-.008	.133	.039	.121	.161	.223*	.140	.072	.084	.124	.007	-.028	.062	.071	-.083	1	
C21	.026	-.138	-.055	.090	.081	.111	.110	.026	.010	.102	-.066	.010	-.046	-.046	-.059	-.126	-.028	-.043	-.029	.236*	1

C1: Heterostyly; C2: Flower color; C3: Bud color; C4: Anthotaxy diameter; C5: Flower number in an anthotaxy; C6: Peduncle length; C7: Pedicel length; C8: Sepal length; C9: Sepal width; C10: Corolla width; C11: Corolla tube length; C12: Style length; C13: Stigma lobes length; C14: Pistil length; C15: Anther length; C16: Anther width; C17: Leaf length; C18: Leaf width; C19: Petiole length; C20: Fruit length; C21: Fruit width.

* Correlation is significant at the 0.05 level; ** Correlation is significant at the 0.01 level.

**Table 3:** Variance analysis (ANOVA) results of morphologic traits of *L. yunnanensis*.

	Heterostyly	Flower color	Bud color	Anthotaxy diameter	Flower number in an anthotaxy	Peduncle length	Pedicel length
GDX	1.47 ± 0.51a	2.07 ± 0.25bc	2.33 ± 0.48b	16.11 ± 2.55a	36.65 ± 20.63a	2.69 ± 0.49a	0.61 ± 0.10a
GCG	1.62 ± 0.49a	1.48 ± 0.57d	1.79 ± 0.68c	13.89 ± 2.50b	23.62 ± 10.53b	2.23 ± 0.40b	0.58 ± 0.12a
FMM	1.55 ± 0.51a	2.07 ± 0.37bc	2.31 ± 0.54b	15.02 ± 2.22ab	27.38 ± 9.97b	2.17 ± 0.49b	0.47 ± 0.12c
FPK	1.43 ± 0.50a	2.10 ± 0.31b	2.53 ± 0.51ab	13.85 ± 1.98b	23.37 ± 8.03b	2.09 ± 0.52b	0.55 ± 0.10ab
LLO	1.70 ± 0.47a	2.57 ± 0.57a	2.80 ± 0.55a	14.60 ± 3.44b	26.12 ± 9.83b	2.02 ± 0.60bc	0.48 ± 0.12c
LCM	1.43 ± 0.50a	1.83 ± 0.46d	2.40 ± 0.62ab	12.03 ± 1.71c	14.76 ± 5.61c	1.78 ± 0.39c	0.51 ± 0.13bc
	Sepal length	Sepal width	Corolla width	Corolla tube length	Style length	Stigma lobes length	Pistil length
GDX	1.48 ± 0.18b	0.39 ± 0.06a	4.36 ± 0.56a	3.24 ± 0.24a	3.16 ± 0.42a	0.64 ± 0.21ab	3.17 ± 0.47a
GCG	1.47 ± 0.16b	0.35 ± 0.04a	4.36 ± 0.46a	3.22 ± 0.29a	3.20 ± 0.46a	0.70 ± 0.21ab	3.12 ± 0.46ab
FMM	1.60 ± 0.19a	0.37 ± 0.05a	4.51 ± 0.36a	3.23 ± 0.23a	3.23 ± 0.46a	0.66 ± 0.21ab	3.16 ± 0.51a
FPK	1.44 ± 0.16b	0.37 ± 0.08a	3.93 ± 0.42b	3.25 ± 0.37a	3.14 ± 0.39a	0.76 ± 0.26a	3.26 ± 0.56a
LLO	1.45 ± 0.32b	0.36 ± 0.09a	3.93 ± 0.81b	2.98 ± 0.60b	3.32 ± 0.79a	0.62 ± 0.24b	2.85 ± 0.67b
LCM	1.43 ± 0.22b	0.37 ± 0.06a	4.03 ± 0.41b	3.19 ± 0.34b	3.15 ± 0.45a	0.74 ± 0.2ab	3.23 ± 0.60a
	Anther length	Anther width	Leaf length	Leaf width	Petiole length	Fruit length	Fruit width
GDX	0.52 ± 0.04a	0.12 ± 0.01cd	16.97 ± 2.20a	4.84 ± 0.70a	1.42 ± 0.23ab	1.66 ± 0.23ab	0.85 ± 0.06a
GCG	0.59 ± 0.07a	0.14 ± 0.02a	14.64 ± 2.42b	4.42 ± 0.81b	1.40 ± 0.27b	1.59 ± 0.25ab	0.78 ± 0.30a
FMM	0.55 ± 0.05a	0.12 ± 0.02bc	15.70 ± 1.78b	4.57 ± 0.61ab	1.54 ± 0.26a	1.50 ± 0.16b	0.79 ± 0.07a
FPK	0.56 ± 0.05a	0.13 ± 0.03b	14.60 ± 1.27b	4.22 ± 0.56b	1.37 ± 0.20b	1.57 ± 0.17ab	0.80 ± 0.07a
LLO	0.48 ± 0.10a	0.11 ± 0.02d	14.52 ± 3.69b	4.37 ± 1.11b	1.30 ± 0.45b	1.76 ± 0.52a	0.78 ± 0.07a
LCM	0.49 ± 0.04a	0.11 ± 0.01d	11.56 ± 2.29c	3.45 ± 0.62c	1.01 ± 0.22c	1.61 ± 0.16ab	0.80 ± 0.06a

The same small-letter means no significant difference in the same column, *p* < 0.05.

**Table 4:** Variation coefficients, variance components and phenotypic differentiation coefficients of phenotypic traits of *L. yunnanensis* populations.

	Variation coefficients (%)						Mean	Variance component		Phenotypic differentiation coefficient among populations (%)
	GDX	GCG	FMM	FPK	LLO	LCM		Among population	Within population	
Heterostyly	34.60	30.47	32.62	35.16	27.42	35.16	32.62	0.00	0.25	0.02
Flower color	12.28	38.75	17.95	14.53	22.14	25.15	26.78	0.12	0.19	27.79
Bud color	20.55	37.65	23.43	20.03	19.67	25.89	26.83	0.10	0.32	8.42
Anthotaxy diameter	15.84	18.00	14.75	14.27	23.52	14.21	17.77	2.14	2.82	36.56
Flower number in an anthotaxy	56.30	44.57	36.42	34.36	37.62	38.02	52.58	47.23	47.01	50.24
Peduncle length	18.34	17.84	22.41	25.01	29.79	21.76	25.66	0.14	0.11	63.84
Pedicel length	16.34	20.16	25.28	17.90	25.17	26.15	23.51	0.00	0.01	10.26
Sepal length	11.98	11.09	12.01	10.80	22.33	15.21	14.71	0.00	0.03	0.05
Sepal width	14.88	11.20	13.53	20.57	24.60	16.28	17.50	0.00	0.00	0.16
Corolla width	12.74	10.56	8.07	10.64	20.67	10.19	13.60	0.03	0.17	2.85
Corolla tube length	7.57	8.99	7.11	11.30	20.18	10.57	11.76	0.00	0.07	0.09
Style length	13.35	14.37	14.3	12.46	23.92	14.33	16.00	0.03	0.16	3.15
Stigma lobes length	32.68	30.79	31.8	34.32	39.15	27.10	33.07	0.00	0.04	1.03
Pistil length	14.93	14.63	16.00	17.26	23.46	18.63	17.87	0.02	0.23	0.81
Anther length	8.54	11.26	9.59	9.19	20.64	8.52	13.86	0.00	0.00	20.16
Anther width	6.24	16.34	15.06	19.27	19.59	6.57	17.50	0.00	0.00	6.57
Leaf length	12.95	16.5	11.33	8.70	25.41	19.76	19.62	3.76	2.56	68.35
Leaf width	14.37	18.34	13.37	13.29	25.30	17.86	20.03	0.24	0.26	45.38
Petiole length	15.97	19.50	16.89	14.79	34.51	21.95	24.36	0.03	0.04	33.49
Fruit length	13.96	15.82	10.48	10.66	29.79	10.20	18.10	0.01	0.02	4.60
Fruit width	6.96	38.47	9.05	8.33	8.56	7.00	13.42	0.00	0.00	8.72
Mean	17.21	21.20	17.21	17.28	24.93	18.60	21.72	/	/	18.69

### 3.2 Genetic diversity

The 17 pairs of SSR primers developed previously by Zhang et al. (2022) were used for the genetic diversity analysis of 164 individuals from six *L. yunnanensis* populations (**Supplementary Figure S1**), a total of 74 alleles were detected and the average number of alleles per locus was 4.35. The average of the polymorphic information content (PIC) was 0.356 (**Table 5**). According to the polymorphism evaluation criteria of Botstein: PIC > 0.5 was highly polymorphism, 0.5 > PIC > 0.25 was moderately polymorphism, PIC < 0.25 was low-grade polymorphism (Botstein et al., 1980; Wang et al., 2014). 17 primer pairs were highly or moderately polymorphisms, which could be used to analyze the genetic diversity of *L. yunnanensis*.

**Table 5:** Genetic diversity information of 17 polymorphic EST-SSR markers in *L. yunnanensis*.

Locus	Size range (bp)	Na	Ne	I	Ho	He	Fis	Fst	PIC
N3	261-279	4	2.292	0.964	0.643	0.562	-0.144	0.025	0.513
N6	212-228	5	1.840	0.715	0.385	0.404	0.047	0.163	0.413
N9	127-187	8	2.092	0.822	0.563	0.498	-0.132	0.110	0.456
N10	194-224	2	1.423	0.353	0.306	0.239	-0.278	0.188	0.246
Z13	117-132	5	1.610	0.671	0.450	0.360	-0.248	0.047	0.346
N22	165-180	4	2.356	0.937	0.556	0.570	0.024	0.028	0.519
N24	268-280	4	1.153	0.234	0.090	0.114	0.215	0.072	0.125
Z31	228-240	5	2.098	0.855	0.509	0.459	-0.108	0.251	0.580
Z32	114-122	3	1.215	0.314	0.102	0.166	0.387	0.038	0.163
Z33	255-270	5	1.277	0.444	0.131	0.215	0.393	0.017	0.211
Z36	211-219	3	1.643	0.551	0.397	0.327	-0.214	0.163	0.354
Z38	109-118	4	1.422	0.520	0.343	0.285	-0.204	0.051	0.285
N41	197-221	6	1.146	0.248	0.088	0.114	0.232	0.052	0.119
Z48	215-225	6	1.576	0.631	0.335	0.323	-0.035	0.075	0.328
Z50	144-154	5	2.486	1.066	0.914	0.593	-0.543	0.026	0.548
Z54	212-216	2	1.509	0.487	0.298	0.317	0.061	0.354	0.372
X70	244-250	3	1.939	0.716	0.416	0.433	0.041	0.244	0.479
Mean	/	4.4	1.710	0.619	0.384	0.352	-0.091	0.125	0.356

Na, number of alleles; Ne, effective number of alleles; I, Shannon’s information index; Ho, observed heterozygosity; He, expected heterozygosity; Fis, inbreeding coefficients; Fst, genetic differentiation coefficient; PIC, polymorphism information content.

For six populations of *L. yunnanensis*, number of alleles (*Na*), effective number of alleles (*Ne*), Shannon’s information index (*I*), observed heterozygosity (*Ho*), expected heterozygosity (*He*) ranged from 2.294 to 3.588, from 1.513 to 1.827, from 0.449 to 0.749, from 0.335 to 0.476 and from 0.269 to 0.426, respectively (**Table 6**). The populations with the highest levels of genetic diversity were LLO (*Ho* = 0.476 and *He* = 0.426) and LCM (*Ho* = 0.424 and *He* = 0.381), and the lowest was GDX (*Ho* = 0.335 and *He* = 0.269).In addition, LLO, LCM, FMM and FPK populations all had private alleles (*P_AS_*). The LCM and LLO populations had not only the highest genetic diversity but also the largest number of private alleles, indicating that the two populations in Lushui County were unique natural populations.

**Table 6:** Genetic diversity of *L. yunnanensis* populations based on EST-SSR makers.

Population	GDX	GCG	FMM	FPK	LLO	LCM	Mean/Total
Sample size	29	26	29	26	30	24	164 (Total)
*Na*	2.294	2.588	3.176	3.000	3.529	3.588	3.029 (Mean)
*Ne*	1.513	1.713	1.702	1.743	1.827	1.756	1.710 (Mean)
*Ho*	0.335	0.342	0.353	0.373	0.476	0.424	0.384 (Mean)
*He*	0.269	0.345	0.348	0.341	0.426	0.381	0.352 (Mean)
*I*	0.449	0.594	0.629	0.611	0.749	0.683	0.619 (Mean)
*Fis*	-0.243	0.010	-0.014	-0.093	-0.118	-0.112	-0.095 (Mean)
*P_AS_*	0	0	1	2	1	4	10 (Total)

Na, number of alleles; Ne, effective number of alleles; Ho, observed heterozygosity; He, expected heterozygosity; I, Shannon’s information index; Fis, inbreeding coefficients; P_AS_, private alleles.

### 3.3 Genetic differentiation

Genetic differentiation coefficient (*F_ST_*) and gene flow (*Nm*) of six *L. yunnanensis* populations were 0.071 and 4.368, respectively (**Table 7**), indicating that the intensity of gene flow was very high and the degree of genetic differentiation was not significant among populations. The results of *F*-statistics in each locus are shown in **Table 5**. The results indicated that the inbreeding coefficients (*Fis*) of most loci were less than zero with an average of - 0.091, which indicates a great excess of heterozygosity in this species. At the species level, the AMOVA revealed that the genetic variation was mainly distributed within populations (86.96%), and the distribution of genetic variation among populations accounted for only 13.04% (**Table 8**). At the group level, higher genetic variation was observed within populations than among populations in both geographical groups (**Table 8**). These results were consistent with the results of genetic differentiation coefficient and gene flow analysis. The genetic variation within the populations from the group 2 (95.68%) was higher than that within the populations from the group 1 (88.66%) (**Table 8**). There was high genetic differentiation between the two groups (*F_ST_* = 0.162). The southernmost population LCM and the northernmost population GDX had the greatest genetic differentiation and the least gene exchange. LLO, LCM and FPK populations (group 2) distributed in the southern region of the Nujiang River, the *Nm* between every two populations was greater than 7.000, which illustrates the gene communication between these three populations were very frequent.

**Table 7:** Gene flow (above) and genetic differentiation coefficient (below) among *L. yunnanensis* populations.

Population	GDX	GCG	FMM	FPK	LLO	LCM
GDX	***	2.979	3.061	2.050	2.705	1.847
GCG	0.077	***	12.410	2.433	3.056	2.435
FMM	0.076	0.020	***	2.874	3.528	2.763
FPK	0.109	0.093	0.080	***	7.480	8.466
LLO	0.085	0.076	0.066	0.032	***	7.431
LCM	0.119	0.093	0.083	0.029	0.033	***

*** means no value.

**Table 8:** **Analysis of molecular variance (**AMOVA) results for six populations of *L. yunnanensis*.

Type	Source of Variation	d.f.	Sum of Squares	Variance Component	Percentage of Variation	Fixation Index
Whole	Among populations	5	139.876	0.457 Va	13.04	FST = 0.130
	Within populations	322	980.664	3.046 Vb	86.96	
	Total	327	1120.540	3.502		
Group1	Among populations	2	45.138	0.354 Va	11.34	FST = 0.113
	Within populations	165	456.910	2.769 Vb	88.66	
	Total	167	502.048	3.123		
Group2	Among populations	2	22.665	0.151 Va	4.32	FST = 0.043
	Within populations	157	523.754	3.336 Vb	95.68	
	Total	159	546.419	3.487		
Two groups	Among groups	1	72.073	0.336 Va	9.23	FST = 0.162
	Among populations within groups	4	67.803	0.255 Vb	7.01	FSC = 0.077
	Within populations	322	980.664	3.046 Vc	83.75	FCT = 0.092
	Total	327	1120.540	3.636		

Whole: the analysis included all collected populations as one hierarchical group. Group 1: the analysis included populations distributed in the northern region of the Nujiang River (GCG, GDX and FMM). Group 2: the analysis included populations distributed in the southern region of the Nujiang River (FPK, LLO and LCM). Two groups: the analysis included two geographical groups (Group 1 and Group 2).

### 3.4 Genetic identity and genetic distance between population

Genetic identity (GI) and genetic distance (GD) can measure genetic relationships between populations in terms of similarity and difference, respectively. As can be seen from **Table 9**, the highest GI (0.982) and lowest GD (0.018) were found between the GCG and FMM populations, indicating that the two populations had a close phylogenetic relationship. In contrast, the minimum GI (0.850) with maximum GD (0.163) was found between LCM and GDX populations, indicating that the degree of differentiation between the two populations was higher than that of other populations. The results of genetic identity and distance analysis were consistent with the analysis results of *F_ST_* and *Nm*.

**Table 9:** Genetic identity (above) and genetic distance (below) among *L. yunnanensis* population.

Population	GDX	GCG	FMM	FPK	LLO	LCM
GDX	***	0.912	0.906	0.882	0.908	0.850
GCG	0.092	***	0.982	0.879	0.896	0.867
FMM	0.099	0.018	***	0.889	0.904	0.874
FPK	0.126	0.129	0.118	***	0.966	0.964
LLO	0.096	0.109	0.101	0.035	***	0.952
LCM	0.163	0.142	0.134	0.037	0.050	***

*** means no value.

### 3.5 Cluster and principal component analysis

**Figure 3:**
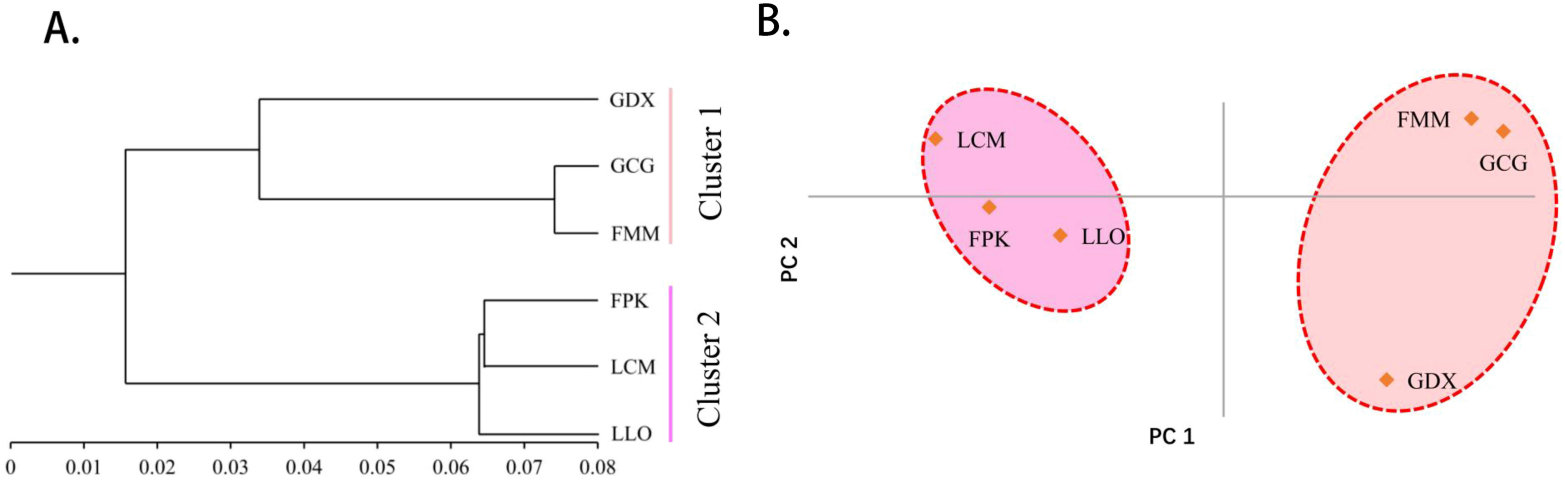
The UPGMA cluster dendrogram **(A)** and Principal component analysis **(B)** of six *L. yunnanensis* populations based on genetic distance.

As can be seen from the UPGMA cluster result (**Figure 3A**), the six populations were also divided into two clusters. GDX, GCG and FMM were clustered into one cluster, the remaining three populations gather into the other cluster. According to the results of principal component analysis (**Figure 3B**), the three populations on the left of the axis, FPK, LLO and LCM, clustered into one category. The three populations on the right of the axis, GDX, GCG and FMM, are grouped into one group. The results of principal component analysis were consistent with those of cluster analysis.

**Figure 4:**
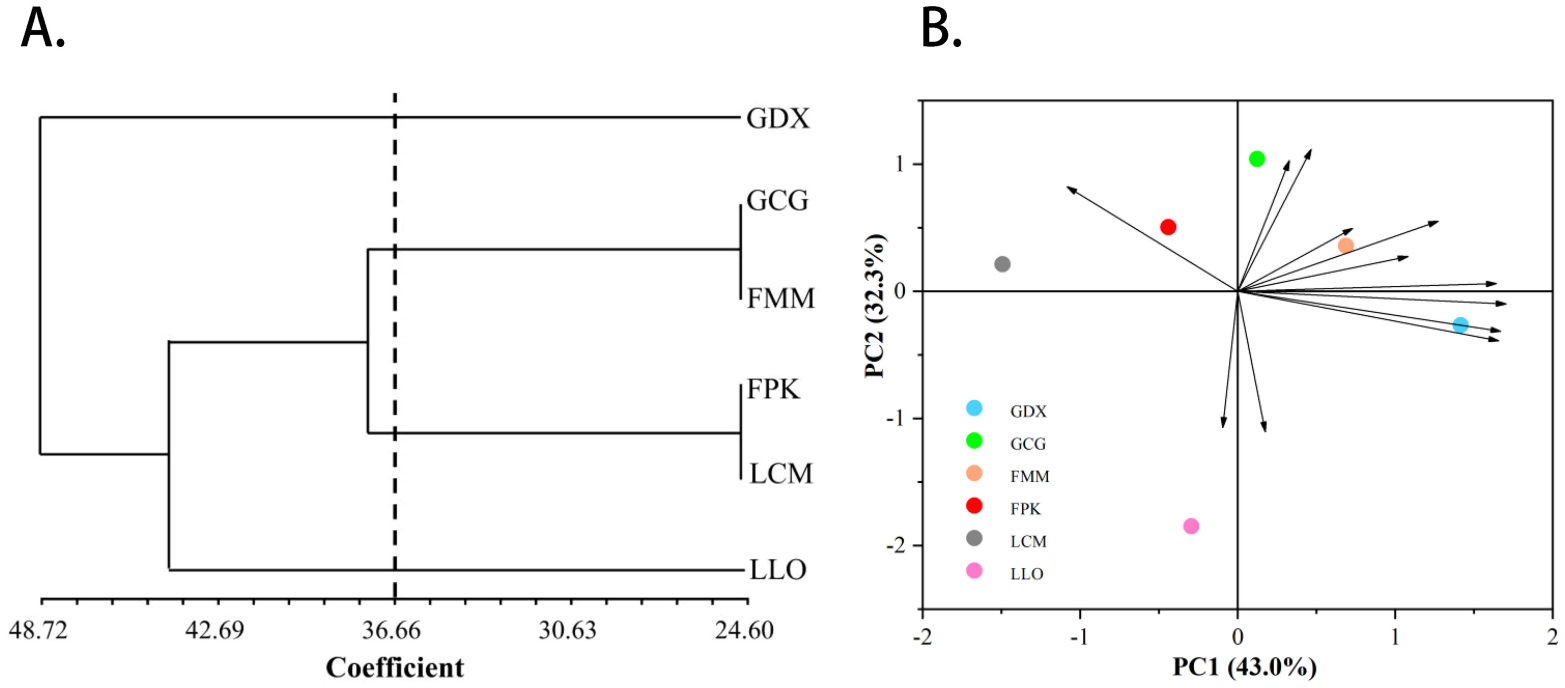
The UPGMA cluster dendrogram **(A)** and Principal component analysis **(B)** of six *L. yunnanensis* populations based on morphological traits.

In addition, we also conducted principal component and cluster analysis on phenotypic traits (**Figure 4**), and the main phenotypic indicators of *L. yunnanensis* resources could be determined through the results of principal component analysis. The ANOVA results of the morphological traits showed that five traits have no significant difference between the *L. yunnanensis* populations, and the correlation test showed that there were high correlations between some traits. Based on this, we removed traits that were not significantly different between populations and traits that highly correlated. Principal component analysis was performed on the remaining 12 morphological traits, and the three principal components with eigenvalues greater than 1 were extracted for analysis. The cumulative contribution rate of the three principal components reached 90.67%, representing most of the information of the original variables. The contribution rate of principal component 1 was 43.04%, and the eigenvectors absolute value of anthotaxy diameter, flower number in an anthotaxy, leaf length and peduncle length were relatively large. The contribution rate of principal component 2 was 32.32%, among which the eigenvectors absolute value of flower color, corolla tube length, fruit length and anther width were larger. The contribution rate of principal component 3 was 15.32%, in which pedicel length and sepal length played the main role. Among the phenotypic traits, anthotaxy diameter, flower number in an anthotaxy, leaf length, peduncle length, flower color, corolla tube length, fruit length and anther width contributed more to the phenotypic trait variation, that is, they had the greatest impact on the ornamental value of *L. yunnanensis*. The results of cluster analysis showed that the six populations could be grouped into three major groups. GDX is a separate class; GCG and FMM population are grouped into group A, FPK and LCM population are grouped into group B, and group A and B are grouped together into one class. The LLO population is also a separate class. The results of the cluster and the principal component analysis were basically consistent.

### 3.6 Population structure analysis

**Figure 5:**
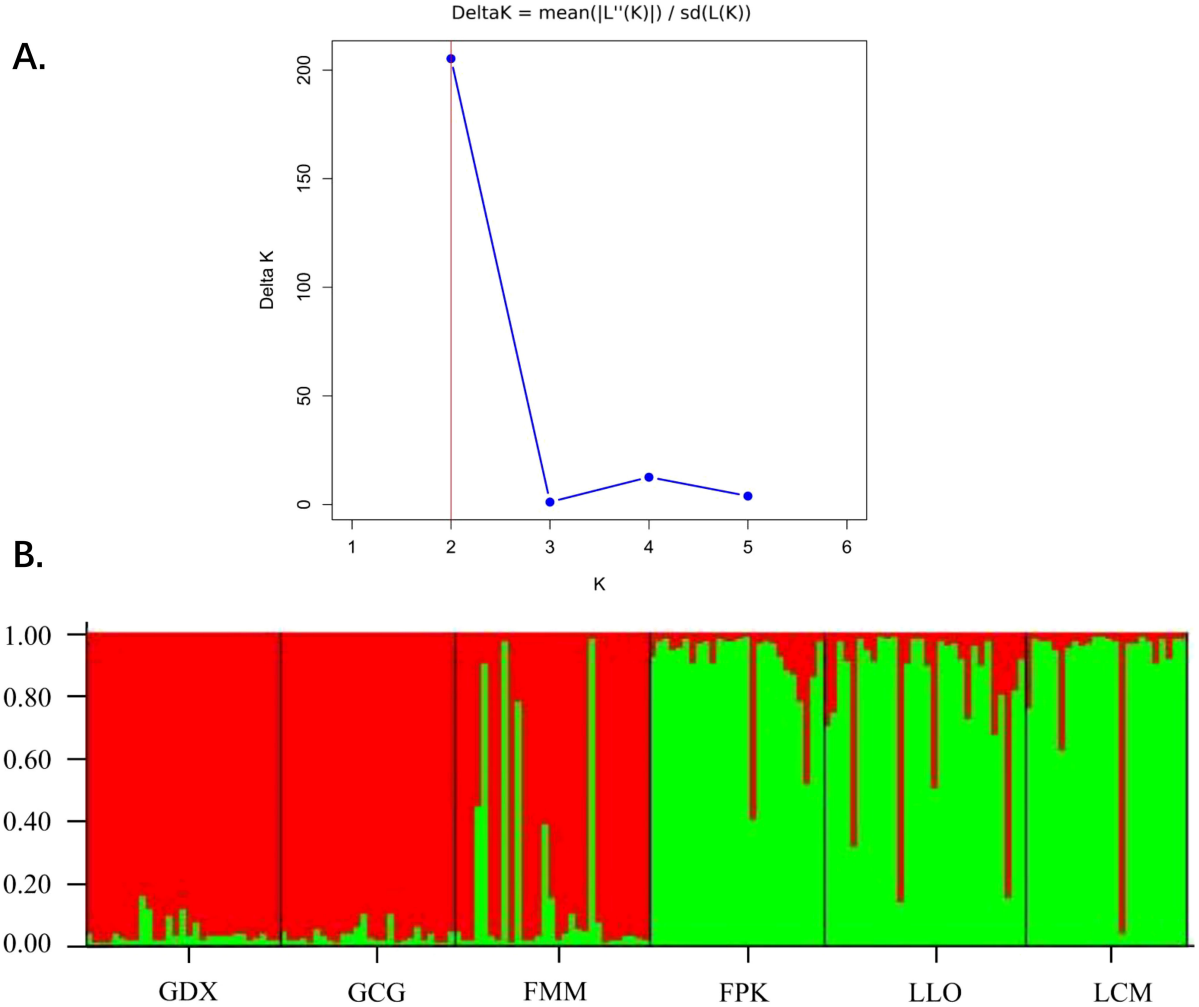
Population structure analysis of six *L. yunnanensis* populations. **(A)** Determination of the optimal K value based on the ΔK estimation; **(B)** Estimated genetic clustering (K=2) obtained with the STRUCTURE program for six populations of *L. yunnanensis*; each color represents a different cluster.

Based on STRUCTURE analysis, the result showed that a ΔK is at the maximum when K=2 (**Figure 5A**). This indicated that 164 individuals of six populations can be assembled in two groups, which were shown as red and green colors in **Figure 5B**. As can be seen from the figure, GDX, GCG and FMM distributed in the northern region of the Nujiang River were closely related, while FPK, LLO and LCM distributed in the southern region of the Nujiang River were closely related, suggesting a relationship between genetic structure and geographical distribution of the *L. yunnanensis* populations.

## 4 Discussion

### 4.1 Morphological characteristics of *L. yunnanensis* population

Phenotypic traits are the sum of traits retained by plants after long-term adaptation to a certain environment, and are the result of the interaction between plant genes and the environment. Therefore, description and evaluation based on plant phenotypic traits is one of the direct and effective methods to study genetic variation and diversity of plant resources (Stepansky et al., 1999). There are usually different degrees of correlation between traits in plants, based on which the status of one trait can be predicted by another trait, so that the highly correlated traits can be removed to simplify the process of germplasm evaluation and phenotype observation (Zhou et al., 2024; Zhu et al., 2024). In this study, the correlation analysis of 21 morphological traits revealed that most of the traits had significant or highly significant correlations between each pair, and the relationships among the traits were strong and complex, with overlapping trait information, which also reflected the necessity of principal component analysis. Principal component analysis (PCA) is based on the interrelationships among indicators, and utilizes the method of dimensionality reduction to convert several major phenotypic traits into a few major components, so as to clearly showing the important level of each morphological trait in *L. yunnanensis*. We screened out the main eigenvectors including anthotaxy diameter, flower number in an anthotaxy, leaf length, peduncle length, flower color, corolla tube length, fruit length and anther width by PCA. Therefore, plants that show abnormalities in these indicators need to be focused on, which may be potential breeding materials with special phenotypic traits. At the same time, these indexes can be preferred to evaluate the variation degree of phenotypic traits in the future resource survey of Luculia plants. In this study, we sampled the GDX, GCG, FMM, FPK, LLO, and LCM population from north to south. From the clustering results, we found that the northernmost GDX population was separately clustered into one group, and it was significantly larger than the other five populations in several important morphological traits (anthotaxy diameter, flower number in an anthotaxy, leaf length and peduncle length) that have a strong influence on the ornamental value of *L. yunnanensis*. In terms of geographical distribution, the GDX population is located in the west of Gaoligong Mountain, while the other populations are all located in the east of Gaoligong Mountain, and the geographical isolation is obvious. We speculated that this may be the reason that the clustering results of phenotypic traits cluster the GDX population into one class alone. In addition, from the results of clustering based on phenotypic traits, we found that six populations did not cluster from north to south according to geographical location, among which LLO population showed abnormal phenotypic traits, and the phenotypic traits of LLO population were quite different from those of LCM and FPK population which were geographically close to it. From the geographical location analysis, the altitude of the LLO population was significantly higher than that of the other five populations, so it is speculated that altitude factor may be responsible for the discontinuity of phenotypic trait variation among populations. The results of field investigation also found that the flower color of the *L. yunnanensis* becomes redder with the increase of altitude. Compared with other populations, LLO population located at high altitude had redder flower colors and smaller anther length, corolla width and corolla tube length, which may be caused by the decrease of daily average temperature and the increase of temperature difference between day and night with the increase of altitude. This is consistent with the results of the study on *Saussurea stella* (Wang et al., 2012), *Bergenia purpurascens* (Wang et al., 2010), and *Saussurea macrota* Franch (Chen and Wang, 2014), that is, with the increase of altitude, the plants invest less in vegetative and reproductive organs.

The diversity of phenotypic traits comes from differences in inherited genes and habitats (Cynthia and Johanna, 2004). The degree of phenotypic diversity within populations is lower than among populations, which is often the result of plant adaptation to the environment. The degree of variation and differentiation among populations is closely related to the degree of adaptation of the species to the environment. The phenotypic differentiation coefficient of a species is larger, indicating that it has the greater the ability to adapt to the environment (Pang and Jiang, 1995). The differentiation coefficient among the *L. yunnanensis* populations was relatively small, and the field survey found that it was rarely distributed outside Nujiang Prefecture, Yunnan province, which also indicated that the *L. yunnanensis* had higher requirements on the environment. This is similar to the results of the study on *Rosa platyacantha* (Yang et al., 2013), *Aquilaria sinensis* (Zhao and Zhao, 2007) and *Cerasus campanulate* (Chen, 2008).

### 4.2 Genetic diversity of *L. yunnanensis* population

Research on genetic diversity may contribute insight into the origin and evolution of species, so as to offer help for the effective conservation and further utilization of plant genetic resources, and provide theoretical basis for the formulation of plant breeding programs and endangered species protection strategies (Li et al., 2020; Aleksandra et al., 2021; Yu et al., 2021; An et al., 2024). Nybom analyzed the genetic diversity of plant populations with codominant markers and used *He* to make statistics. According to the differences of breeding system and geographic range, the mean of *He* among outcrossing plants and endemic species was 0.65 and 0.42, respectively (Nybom, 2004). However, the *He* of six *L. yunnanensis* populations was 0.352, which was lower than the average *He* of outcrossing plants and endemic species. And compared our results with the *Luculia pinceana* of same genus, the *He* of *L. yunnanensis* was lower than that of *L. pinceana* (*He*=0.555) (Zhou, 2011). The reason for this result may be related to the relatively limited geographical distribution area of the species. Generally, species with a wide range of natural distribution tend to have higher genetic diversity than those with a small distribution range (Man-Kyu and Hong-Wook, 2000). The genetic diversity results of the six *L. yunnanensis* populations showed that the populations located in the south had higher genetic diversity than those located in the north. Our previous field survey results showed that *Luculia pinceana* was the main dominant species in the south of the LCM population, and *L. yunnanensis* had never been found in this area. In addition, Wan had demonstrated cross-compatibility between the two species (Wan, 2019). Therefore, we speculated that the genetic diversity of populations in the southern part was higher than that in the northern part of the distribution area, which may be related to the natural interspecific hybridization between *L. yunnanensis* and *L. pinceana* in the southern part. But this inference needs to be confirmed by further studies.

### 4.3 Genetic structure and genetic relationship of *L. yunnanensis* population

Genetic differentiation coefficient (*F_ST_*) is an index reflecting genetic differentiation or inbreeding coefficient between populations, and can be used to evaluate genetic diversity among populations (Campbell and Dooley, 1992). According to Wright’s evaluation criteria for *F_ST_*, 0.25 > *F_ST_* > 0.15 was highly genetic differentiation, 0.15 > *F_ST_* > 0.05 was moderately genetic differentiation, *F_ST_* < 0.05 was low-grade genetic differentiation (Wright, 1922). Gene flow (*Nm*) refers to the exchange and transfer of genetic material within and between populations. When *Nm* > 1, it represents the gene flow between populations is greater (Hamrick and Godt, 1996). Generally, the greater the degree of genetic differentiation, the weaker the gene flow, that is, a lower gene migration rate among populations (Waples, 1998; Raaijeveld-Smit et al., 2005; Gerlach et al., 2010; Schmidt et al., 2013). In this study, the *F_ST_* of six *L. yunnanensis* populations ranged from 0.020 to 0.119, with an average of 0.071 (0.15 > *F_ST_* > 0.05), indicating that low-grade or moderate genetic differentiation between the two populations. The results of AMOVA analysis also confirmed this point, that is, the genetic variation of *L. yunnanensis* was mainly distributed within populations. This was consistent with the results of Zhou study on genetic diversity of 25 *L*. *pinceana* populations using seven pairs of SSR primers (Zhou, 2011), namely, the genetic variation was mainly concentrated within populations rather than among populations, the source of variation among populations was relatively little. In addition, the Nm results showed that the gene communication among the three populations distributed in the southern region of the Nujiang River (Group 2) was very frequent, and the differentiation degree was small. It can also be found from the AMOVA results that the *F_ST_* among the group 2 populations was 0.043 (*F_ST_* < 0.05), indicating a low-grade genetic differentiation among these three populations.

Cluster analysis and population genetic structure analysis are effective methods to research on genetic diversity of germplasm resources. The analysis of population genetic structure can reflect the gene exchange and infiltration among populations, which is helpful for breeders to accurately grasp the genetic relationship among germplasms (Alavi-Siney et al., 2022; Bakɪr et al., 2022). In our study, the results of UPGMA clustering, PCA and STRUCTURE analysis were completely consistent. The results showed that the populations of GDX, GCG and FMM were divided into one group, and the FPK, LLO and LCM were divided into another group. It could be seen that there was an obvious geographical division of the *L. yunnanensis* populations. One group (GDX, GCG and FMM) distributed in the northern region of the Nujiang River, and the other group (FPK, LLO and LCM) distributed in the southern region of the Nujiang River. By analyzing the genetic differentiation coefficient, gene flow, genetic identity and genetic distance among the populations, it was found that the gene exchange among southern populations were very frequent, the genetic distance were small. We analyzed the reasons for the formation of genetic structure among *L. yunnanensis* populations in combination with the geographical environment of Nujiang prefecture where the sample was collected. In Nujiang Prefecture, from west to east are the Dandalika Mountain, Dulong River, Gaoligong Mountain, Nujiang River, Biluo Snow Mountain, Lancang River, Yunling Mountain. Four mountains and three rivers are arranged alternately, forming three Grand Canyons (Wang et al., 2000). If Gaoligong Mountain is regarded as the geographical dividing line, the six populations can be divided into two parts, among which the GDX population is located in Dulong River Gorge, and the other five populations are distributed in Grand Canyon of Nujiang River. If the Nujiang River is regarded as a geographical dividing line, the six populations can be divided into two parts: GDX, FMM, LLO and LCM populations are located in the west of the Nujiang River, and GCG and FPK populations are located in the east of the Nujiang River. Based on the flow direction of the Nujiang River, the populations of GDX, GCG and FMM are located in the northern region of the Nujiang River, while LLO, FPK and LCM are located in the southern region of the Nujiang River. GDX and other populations are separated by Gaoligong Mountain with more than 5000 meters above sea level, and we speculate that this is one of the reasons for the low gene exchange and the low possibility of inbreeding between GDX and other populations. LLO and FPK populations are separated by the Nujiang River, but have the smallest geographical distance and can be seen across the river. The results showed that the genetic differentiation between these two populations was very small, suggesting that the Nujiang River did not play a role in isolating the gene exchange among the populations of *L. yunnanensis*.

In addition to geographical distance, the genetic structure of a plant species is also determined by its own reproductive characteristics (Guo et al., 2011; Song et al., 2013). Although there is a lack of research on the reproductive system of *L. yunnanensis*, Zhou found in his study on plants of the same genus that only monomorphic populations of *L. pinceana* had high self-compatibility and low outcrossing rate, while others were self-incompatibility (Zhou, 2011). In the field investigation, we did not find monomorphic populations of *L. yunnanensis*, so it is speculated that *L. yunnanensis* is mainly outcrossed and may be mainly insect-pollinated like *L. pinceana*. Pollinators play an important role in gene exchange among populations. The main pollinators of the *Luculia* are *Apis florae*, insects of *Vespa* and *Bombus* (Ma et al., 2009). *Apis florae* could be active within a radius of 2~3 km (Beekman and Ratnieks, 2000; Abrol, 2006), which could fly up to 10 km (Vaissière et al., 1984; Fan and He, 2010); and the maximum range of wasps also could reach 10 km (Li, 2017). This also explains why inbreeding between FPK and LLO, and between GCG and FMM, was not prevented by the Nujiang River.

Combined with the previous studies, the characteristics of high seed setting rate per plant, platysperm and small 1000-grain weight were also found. At the same time, it was found in the field investigation that the *L. yunnanensis* mostly grows in areas with high relative humidity and good ventilation, such as roadside, stream side, forest edge and so on. So, we hypothesized that the *L. yunnanensis* spread its seeds by the wind like the *L. pinceana* (Zhou, 2011). The Grand Canyon of Nujiang River was mainly dominated by north-south wind (You et al., 2007), which assisted the spread of *L. yunnanensis* in the north-south orientation. This was one of the reasons for the gene exchange between the northern and southern populations of Nujiang River was very frequent.

### 4.4 Conservation suggestion of *L. yunnanensis*

In our field resource investigation, we found that (1) the distribution range of *L. yunnanensis* was narrow and its ability to adapt to the environment was limited, (2) human disturbances had led to serious habitat patchiness, and (3) the number of seedlings was very small, some populations had no seedlings, and the natural regeneration ability was weak (Li, 2017). Based on the resource status and genetic diversity results, we put forward some tentative ideas on the conservation measures of *L. yunnanensis*.(1) Our population genetic analysis revealed that the two populations distributed in Lushui County (LLO and LCM) not only exhibits high levels of genetic diversity and gene flow, but also has a high number of private alleles. In addition, *L. yunnanensis* is an ornamental plant, phenotypic traits need to be taken into account when developing conservation measures. The results of phenotypic traits showed that the LLO population had a higher phenotypic variation coefficient than the other five populations, and its flower color was redder than the other populations. Therefore, we recommend that the LLO and LCM populations be considered as independent genetic units and given priority for conservation. (2) The marginal populations represent special germplasm resources that are characterized by low genetic diversity and high levels of genetic differentiation relative to the other populations. The GDX population isolated by Gaoligong Mountain is a marginal population, its genetic diversity is lowest and the degree of differentiation between it and other populations is relatively high. Moreover, in terms of morphological traits, some important traits such as the anthotaxy diameter and the flower number in an anthotaxy of GDX were significantly larger than those of other populations. So, GDX should also be regarded as an independent unit for key conservation. Due to the high ornamental value of *L. yunnanensis* and the weak protection awareness of local residents, its wild population had been damaged to varying degrees. At present, there is a lack of research data on *L. yunnanensis*, so we should strengthen the research work on it, which can not only provide scientific advice on the protection and utilization of *L. yunnanensis* resources, but also improve its importance and avoid its resources being further broken.

## 5 Conclusions

This article used morphological traits and EST-SSR markers as a research tool to comprehensively evaluate the genetic diversity and structure of six *L. yunnanensis* populations collected from the species that only existing in three counties, and proposed some conservation strategies for this vulnerable species. This study is the first comprehensive report of the genetic diversity of natural *L. yunnanensis* populations. The genetic diversity results of the six *L. yunnanensis* populations showed that the populations located in the south had higher genetic diversity than those located in the north. The LLO and LCM populations distributed in Lushui County had the highest genetic diversity. The GDX populations isolated by Gaoligong Mountain had the lowest genetic diversity, and the degree of differentiation between it and other populations is relatively high. These populations also exhibit significant differences from other populations in several important morphological traits. And the results of phenotypic and genetic variation analysis were consistent, indicating that the most of variation exists within population. In addition, genetic structure of *L. yunnanensis* populations identified is consistent with the geographical distribution of these populations, the populations distributed in the southern region and distributed in the northern region of the Nujiang River clustered into one group respectively. Combining the results of this study and resource status, we recommend that give priority to the protection of LLO, LCM and GDX population. The findings of this study have provided new ideas and guidance for the rational development, utilization and protection of *L. yunnanensis*, and they will also provide a scientific basis for the ecological restoration and economic development in Nujiang River basin.

## Data Availability

The original contributions presented in the study are included in the article/**Supplementary Material**. Further inquiries can be directed to the corresponding authors.
