# Supplementary material for: Genetic diversity evaluation of Luculia yunnanensis, a vulnerable species endemic to Yunnan, Southwestern China based on morphological traits and EST-SSR markers
Source: Front Plant Sci. 2024 Aug 14;15:1428364. doi: 10.3389/fpls.2024.1428364 (PMC11363074; doi:10.3389/fpls.2024.1428364)
Supplement: Supplementary file 2 [file Table1.docx]

Supplementary Material

The information of 17 EST-SSR primers

| **Primer** | **Primer Sequence (5’-3’)** | **Repeat Motif** | ***Ta* (°C)** | **Size (bp)** |
| --- | --- | --- | --- | --- |
| N3 | CAAATTCGCGCACCAAAACG | (TGGCGT)5 | 52 | 261–279 |
|  | GCTAGAGAGAAAAGGGGCCG |  |  |  |
| N6 | ACTGCGTACCTCTCCCTCTT | (TAATT)5 | 58 | 212–228 |
|  | TCTCTCTCTCTCGGACGGAC |  |  |  |
| N9 | GACCCCAAGTTGGCTGATCA | (TGTTAC)8 | 60 | 127–187 |
|  | AGGGCACTTCTGTCATTTCGA |  |  |  |
| N10 | CTGGTGCACGAGGATTGAGT | (TCAATT)7 | 60 | 194–224 |
|  | GAAGAGTGCCATGGAAACTGC |  |  |  |
| Z13 | CCTCCCATAGCAGCAGCAAT | (ACC)5 | 54 | 117–132 |
|  | AGTAGTATTAATAATGGCTGGAGGT |  |  |  |
| N22 | CGCTTCTGTGTTCGAAACCA | (ACAAC)7 | 60 | 165–180 |
|  | CAAAGCTTCCCGTCAACAGC |  |  |  |
| N24 | CCCACCGAGCAATACCCAAA | (GAAA)5 | 56 | 268–280 |
|  | ACCTTCTCTGTACTCTGCCT |  |  |  |
| Z31 | GCAATCCTACTCGTGCTGGT | (GGC)6 | 54 | 228–240 |
|  | AGCCAAGACTCGGCAGAAAA |  |  |  |
| Z32 | TGCACTCCATAAAAGAAGAAAACACA | (TATT)5 | 53 | 114–122 |
|  | TGCAGTAACTTCGTGCCCTT |  |  |  |
| Z33 | CCCAACCCACCACACAAGT | (TCT)6 | 54 | 255–270 |
|  | AGAGAGGAGGATCGAGGACG |  |  |  |
| Z36 | TCGGGTCCTAGGGCTTTCTT | (CTTT)5 | 54 | 211–219 |
|  | GGCCCTCCTTGAGCATTGAT |  |  |  |
| Z38 | ACCCAAGGAACTCTGTCTCT | (AAT)6 | 53 | 109–118 |
|  | ACACTTTCGTCGTCCTTAGGT |  |  |  |
| N41 | GCCAGAAGGATAGCTTTCGC | (TCCT)5 | 53 | 197–221 |
|  | GGTTTGTGGTGGTTTTTGGGA |  |  |  |
| Z48 | AGGAAGGGCTTGTTTTTAAGGT | (AG)8 | 52 | 215–225 |
|  | GAGCCAATGACGATCCAGCT |  |  |  |
| Z50 | TCTGCTGCATCCAATGTACTGT | (GT)8 | 54 | 144–154 |
|  | CCTGCCATAGGTGCCCATTT |  |  |  |
| Z54 | AGTAAGTGGGTGGAGGTGGT | (TTGA)5 | 59 | 212–216 |
|  | AGGGGCTGATTCTCTAGCGA |  |  |  |
| X70 | AGCTGGAAACTAAAGGTGGAGG | (ATC)5 | 58.5 | 244–250 |
|  | CTCAGTCTGTCAGGCCTGTG |  |  |  |
